# Fusing RGB and Thermal Imagery with Channel State Information for Abnormal Activity Detection Using Multimodal Bidirectional LSTM

**DOI:** 10.1007/978-3-030-69781-5_6

**Published:** 2021-01-28

**Authors:** Nikolaos Bakalos, Athanasios Voulodimos, Nikolaos Doulamis, Anastasios Doulamis, Kassiani Papasotiriou, Matthaios Bimpas

**Affiliations:** 8 Norwegian Computing Center, Oslo, Norway; 9 University of Trento and Fondazione Bruno Kessler, Trento, Italy; 10 Università degli Studi di Genova, Genoa, Italy; 11 IEIIT Institute, Consiglio Nazionale delle Ricerche (CNR), Genoa, Italy; 12 SINTEF A.S., Oslo, Norway; 13 Engineering Ingegneria Informatica S.p.A., Rome, Italy; 14 Instituto Superior de Engenharia do Porto, Porto, Portugal; 15 University of Padua, Padua, Italy; 16 School of Rural and Surveying Engineering, National Technical University of Athens, 15773 Athens, Greece; 17 Department of Informatics and Computer Engineering, University of West Attica, 12243 Athens, Greece; 18 Columbia University, New York, NY 10027 USA

**Keywords:** Abnormal activity detection, Human intrusion, Multimodal data fusion, Bidirectional LSTM, Critical infrastructure monitoring

## Abstract

In this paper, we present a multimodal deep model for detection of abnormal activity, based on bidirectional Long Short-Term Memory neural networks (LSTM). The proposed model exploits three different input modalities: RGB imagery, thermographic imagery and Channel State Information from Wi-Fi signal reflectance to estimate human intrusion and suspicious activity. The fused multimodal information is used as input in a Bidirectional LSTM, which has the benefit of being able to capture temporal interdependencies in both past and future time instances, a significant aspect in the discussed unusual activity detection scenario. We also present a Bayesian optimization framework that fine-tunes the Bidirectional LSTM parameters in an optimal manner. The proposed framework is evaluated on real-world data from a critical water infrastructure protection and monitoring scenario and the results indicate a superior performance compared to other unimodal and multimodal approaches and classification models.

## Introduction

Abnormal activity detection is a research problem that attracts significant interest in the image and video analysis research community (e.g. [9, 10]). Many different techniques have been proposed in the field of computer vision and video analysis, including methods based on trajectory analysis [12], pixel-level processing [11], combined trajectory and low-level analysis [1], background modelling [14], object detection [13] and tracking [15], activity recognition [16], and crowd behavior analysis [17]. Despite the efficacy of such techniques, their dependence on strictly visual information makes them susceptible to occlusions, difficult fields of view and poor illumination circumstances. This limitation has motivated the exploration of vision techniques beyond the visible spectrum. Thermographic data can provide a useful alternative stream of information. Thermal camera sensors are not sensitive to illumination changes [4]; on the other hand, thermal information does not entail texture or color information. Since both RGB and thermal sensing are actually based on visual cues, an interesting idea is to supplement them by additional data that are not limited by the restrictions of visual information (such as occlusions).

Recent studies have indicated that wireless signal reflection can be effectively leveraged to sense human presence. Different kinds of techniques have been described in the literature, including device-free Software Defined Radio (SDR) methods, which process the Received Signal Strength of a transmitted signal. However, the accuracy of such techniques is often not sufficiently high [18]. In contrast, it has been shown that techniques based on commercial off the shelf (COTS) equipment [5] can yield good performance rates in human presence detection, by making use of Channel State Information (CSI) [7].

Moving on from the input modalities to the machine learning models used for abnormal activity detection, it is clear that deep learning techniques, and especially Convolutional Neural Networks (CNN), have been shown to outperform traditional classifiers [1, 6, 16], which is explained by their high representational capabilities. However, one limitation of CNNs is that they cannot inherently capture temporal interdependencies in a bidirectional manner, i.e. from both past and future time instances, which is an important aspect in time series modeling problems.

In this work, we propose a model based on a Bayesian optimized multimodal bidirectional LSTM neural network for abnormal activity detection. Our model harnesses the power of LSTM networks to capture long and short term dependencies, while the backward and forward pass of the bidirectional version of LSTM ensure the consideration of both past and future time instances. Our proposal also includes a Bayesian optimization framework that optimally tunes the parameters of the utilized bidirectional LSTM. Finally, the combination of heterogeneous input modalities, such as RGB and thermal imagery with Channel State Information (CSI) from wireless signal reflection leads to a significantly improved detection performance compared to cases that are solely based on a single information modality.

## Fusion of RGB and Thermal Imagery with Channel State Information

### RGB Imagery

Contrary to traditional abnormal activity detection systems which are usually based on RGB video sequence input, in the work at hand an additional modality is considered, that of thermographic imagery. Visual streams from RGB cameras are initially processed using the object detection module YOLO (You only look once) [13]. YOLO locates spatial bounding areas on the frame and allocates each area a probability for an object class. A Convolutional Neural Network is used for object detection, comprising 24 convolutional layers and 2 fully connected layers. Each image frame is described as a class image *CL*_*RGB*_, having the same size as the initial RGB image, where the *(x,y)* pixel of the RGB image I*(x,y)* is denoted as ${o}_{k,RGB}(x,y)$, in the class in the following way:

1 $${{CL}_{RGB}(x,y)=o}_{k,RGB}(x,y)$$

where k denotes the object with identity k in the object detection module employed.

### Thermal Imagery

Data acquired by thermographic sensors undergo background subtraction [14]. A class label image CLT is extracted, having the same size as the input thermal frame T, where the (x,y) pixel of T is denoted in the class label image as:

2 $${CL}_{T}\left(x,y\right)= {o}_{b,T}\left(x,y\right),b=\{Background, Foreground\}$$

In order to facilitate the subsequent processing steps, the RGB and thermal image frames are resized so as to become of identical sizes, $NxM$. In other words, ${x}_{RGB}(n)\in {R}^{NxM}$ stands for an image, whereby each pixel indicates the object ID that pixel belongs to. In a similar manner, tensor ${x}_{thermal}(n)\in {R}^{NxM}$ denotes the class label image of the thermographic modality.

### Channel State Information

Channel State Information (CSI) can be leveraged for human movement detection using WiFi devices, based on propagation modeling of a signal from the transmitter to the receiver, supporting many subcarriers due to the Orthogonal Frequency Division Multiplexing (OFDM) principle. CSI includes physical attributes of the wireless channel, such as scattering, power decay per distance, fading, shadowing and effects of interference [7], which are measured by the amplitude/phase over all K available subcarriers:

3 $$H\left(n\right)= {[H\left(n,{f}_{1}\right), H\left(n,{f}_{2}\right), ..., H(n,{f}_{k})]}^{T}$$

where $\mathrm{H}\left(\mathrm{n},{\mathrm{f}}_{\mathrm{i}}\right)$ refers to the amplitude and the phase of the i-th subcarrier with central frequency ${\mathrm{f}}_{\mathrm{i}}$.. Therefore, we have that: $\mathrm{H}\left(\mathrm{n},{\mathrm{f}}_{\mathrm{i}}\right)=|\mathrm{H}\left(\mathrm{n},{\mathrm{f}}_{\mathrm{i}}\right)|{\mathrm{e}}^{\mathrm{j\angle H}\left(\mathrm{n},{\mathrm{f}}_{\mathrm{i}}\right)}.$

Usually, $H\left(n\right)$ input data contain noise and are distorted by outliers. For this reason, CSI signals H(n) need to undergo a pre-processing stage. First, outliers are removed using a Hampel identifier [8] or density-based clustering algorithms such as DBSCAN [23]. In the sequel, noise is removed with wavelet denoising, followed by normalization, correlation of subcarriers and, finally, eigenvector processing of the signals. After pre-processing, CSI data are used as input to a linear SVM for human intrusion detection. The SVM’s output classification IDs, say ${C}_{CSI}\left(n\right)$, will be used as input to our proposed multimodal bidirectional LSTM framework. The CSI related input ${x}_{CSI}\left(n\right)$ is given by:

4 $${x}_{CSI}\left(n\right)= {[H\left(n\right){C}_{CSI}\left(n\right)]}^{T}$$

For spatial coherency with the visual input data, tensor ${x}_{CSI}\left(n\right)$ is expanded over the ${R}^{NXM}$ grid, forming an additional input channel.

### Fusion of RGB, Thermal and CSI Modalities

Approaches based on solely one of the above types of information are unavoidably plagued by the limitations of each information modality (e.g. occlusions, noise, etc.). We hereby propose the fusion of the above described information channels to create a multimodal input tensor x(n):

5 $$x\left(n\right)= {[{x}_{RGB}\left(n\right){, x}_{thermal}\left(n\right), {x}_{CSI}(n)]}^{T}$$

where ${x}_{RGB}\left(n\right)$ is the data tensor pertaining to RGB visual signals, ${x}_{thermal}(n)$ the respective data tensor of the thermal component, and ${x}_{CSI}\left(n\right)$ the data tensor pertaining to the WiFi reflection signal.

## Bayesian Optimized Multimodal Bidirectional LSTM

### Bidirectional LSTM

**Fig. 1.**
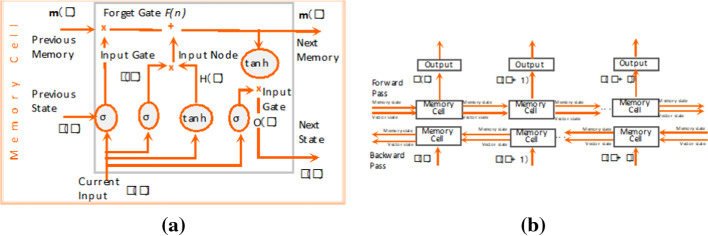
**(a)** The memory cell of a LSTM network. **(b)** Bidirectional LSTM unfolded in time

LSTMs is a type of Recurrent Neural Network (RNN) which was designed to address the problem of exploding and vanishing gradient that can arise when training traditional RNNs. LSTM networks are a good fit to classifying, processing and making predictions based on time series data, since there can be lags of unknown duration between important events in a time series [25–27]. In LSTMs, each node in the hidden layer is replaced by a memory cell, instead of a single neuron [25]. The structure of a memory cell is illustrated in Fig. 1.

The LSTM memory cell is composed of the following: the forget gate, the input node, the input gate, and the output gate. The input gate controls the extent to which a new value flows into the cell, the forget gate controls the extent to which a value remains in the cell and the output gate controls the extent to which the value in the cell is used to compute the output activation of the LSTM unit. The activation function of the LSTM gates is often the logistic sigmoid function.

The goal of the forget gate is to decide what information should be discarded out of the memory cell [24]. The output, denoted as $f\left(n\right)$ ranges between 0 and 1, according to the sigmoid activation function. The forget gate learns whether a previous or future vector state is necessary for the estimation of the current value state. The input node performs the same operation with that of a hidden neuron of a typical recurrent regression model. We denote the output of this node as ${I}_{n}\left(n\right)$. The goal of this node is to estimate the way in which each latent state variable contributes to the final model.

As far as the input gate is concerned, its role is to regulate whether the respective hidden state is sufficiently important. The output of this gate is denoted as ${I}_{g}\left(n\right)$. It has the sigmoid function, therefore its response ranges between 0 and 1. This gate addresses problems related to the vanishing of the gradient slope of a $tanH\left(  \cdot   \right)$ operator. Finally, the output gate regulates whether the response of the current memory cell is sufficiently significant to contribute to the next cell. Therefore, this gate actually models the long range dependency together with the forget gate. The output of this gate is denoted as $O\left(n\right)$.

One of the disadvantages of the memory cell of Fig. 1, is that it considers only past state information. On the contrary, bi-directional forms of LSTM can process data in both directions, and include, therefore, apart from the forward pass an additional backward operation. The structure of a bi-directional LSTM, unfolded in time is presented in Fig. 1(b). Detection of abnormalities in video and CSI time series is an application which can inherently benefit from this additional backward operation of the bidirectional LSTM, which is the base model adopted in this work.

### Bayesian Optimization

We hereby present a Bayesian optimization method for the selection of the bidirectional LSTM model parameters. In lieu of employing manual tuning of model parameters, we hereby present and use a probabilistic Bayesian approach through which model parameters are optimally tuned.

As in all models, let us denote by ${\pi }_{i}$ the set of configurable parameters, e.g. in our case the number of memory cells, the learning rates, etc. Supposing a set Q of different configurations, i.e., ${D}_{1:Q}=\{{\pi }_{1}\dots {\pi }_{Q}\}$, we can then evaluate the error ${\rm E}\left(x,d,\pi \right)$ yielded when (i) the model receives input data x, (ii) its output is compared against the target outputs *d* and (iii) we consider a specific model configuration $\pi$. Let ${E}_{min}$ be the minimum Mean Square Error across all *Q* configurations. The following can then be an improvement function:

6 $$I\left(x,d,\pi \right)=\mathrm{max}\{0, {E}_{min}-E\left(x,d,\pi \right)\}$$

In the sequel, the expectations of Eq. (6) can be computed in a probabilistic context. The probability distribution of the error function for a given set of configurations, $P\left(E|{D}_{1:Q}\right),$ is written in a Bayesian context as:

7 $$P\left(E|{D}_{1:Q}\right)\propto P\left({D}_{1:Q}|E\right)P(E)$$

Usually $P(E)$ follows a Gaussian distribution and $P\left({D}_{1:Q}|E\right)$ is then expressed as a Gaussian process of mean $\mu \left(\pi \right)$ and standard deviation Σ [28]:

8 $$\Sigma =\left[\begin{array}{ccc}k({\pi }_{1},{\pi }_{1})& \dots & k({\pi }_{1},{\pi }_{Q})\\ \vdots & \ddots & \vdots \\ k({\pi }_{Q},{\pi }_{1})& \dots & k({\pi }_{Q},{\pi }_{Q})\end{array} \right]$$

where $k(\bullet )$ is a kernel function. The target of our optimization is to find out a new configuration ${\pi }^{*}\equiv {\pi }_{Q+1}$, which will further reduce the MSE or equivalently increase the improvement $I\left(x,d,{\pi }^{*}\right)$. Then, for the new augmented set ${D}_{1:Q+1}$, that includes ${\pi }^{*}\equiv {\pi }_{Q+1}$, $P{(D}_{1:Q+1}|E)$ will again be a Gaussian process of standard deviation

9 $$\left[\begin{array}{cc}\Sigma & b\\ {b}^{T}& k({\pi }_{Q+1},{\pi }_{Q+1})\end{array}\right]$$

Where ${b=[k(\pi }_{Q+1}, {\pi }_{1})\dots k({\pi }_{Q+1}, {\pi }_{Q})$. Then, according to [28], it can be proven that the ${P(E}_{Q+1}|{{D}_{1:Q},\pi }_{Q+1})$ is also a Gaussian with mean value and standard deviation related with previous variables. Therefore, the new configuration ${\pi }^{*}$ is estimated, which is actually the integral of $I(\bullet )$ and ${P(E}_{Q+1}|{{D}_{1:Q},\pi }_{Q+1})$, that is the probability that $I\left(\bullet \right)$ follows.

## Experimental Evaluation

### Experimental Setup

To scrutinize the effectiveness of the proposed model, we have used a dataset that has been created in the context of the European Horizon 2020 STOP-IT Project (https://stop-it-project.eu/). STOP-IT aims at tackling the protection of critical water infrastructure using novel methods. The dataset includes RGB and thermal video sequences as well as Channel State Information. The RGB data were captured using an OB-500Ae camera with 1280 × 720 pixel resolution at 30 fps. The thermal data were obtained by means of a Workswell InfraRed Camera 640 (WIC) with a 640 × 512 pixel resolution at 30 fps. WiFi data were acquired using a transmitter-receiver couple that comprised a WiFi router (TP-Link N300 TL-WR841N) and an Intel 5300 NIC receiver, with a 0.1 Hz capturing frequency. Data annotation was performed on the basis of pre-determined scenarios by end users that prescribed whether the captured activity over all data modalities should be considered as irregular/abnormal.

The entirety of data across all modalities were normalized so as to be in the same range (0–1). The computer used for all training and testing was an Intel® Core™ i7-6700 CPU@ 4000 GHz CPU with 16GB RAM and an NVIDIA GeForce GTX 1070 with 8GB DDR5 memory. CUDA 9.2 Toolkit was also used for deep learning classifiers.

### Experimental Results

**Fig. 2.**
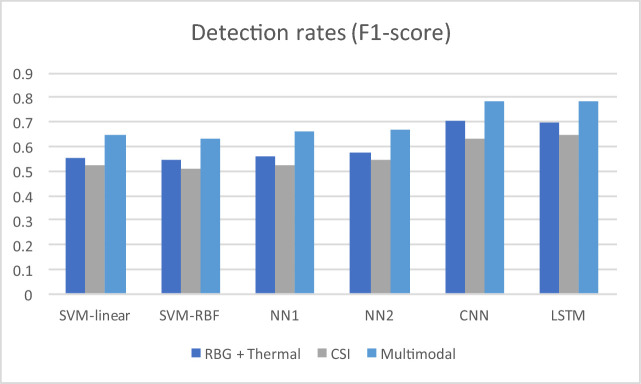
Attained F1-score of shallow and deep learning models for: (i) visual (RGB + thermal), (ii) WiFi-CSI, and (iii) multimodal input.

The first round of experiments focuses on the impact of using fused multimodal data as input, instead of solely considering a single modality. We have initially experimented with the following popular machine learning models: (i) a linear kernel SVM, (ii) a non-linear Radial Basis Function SVM (RBF-SVM), two different architectures of a traditional feedforward neural network: (iii) with 1 hidden layer of 10 neurons/layer and (iv) 2 hidden layers of 10 neurons/layer respectively, (v) a CNN and (vi) a plain LSTM (without bidirectionality or optimization). Fig. 2 depicts the accuracy rates attained by the above classifiers in cases with (a) only RGB and thermal input, (b) CSI (WiFi) and (c) multimodal input. From the results, it is evident that the proposed data fusion scheme of significantly increases the achieved performance detection performance regardless of classification scheme.

In the second round of experiments, we conduct experiments to validate the effectiveness of the proposed multimodal Bayesian optimized bidirectional LSTM. Focusing on the multimodal case, we compare the performance of the proposed model with the six models mentioned above (SVM-linear, SVM-RBF, FNNs, CNN, LSTM). The results of the experiments in terms of precision, recall, F1-score and accuracy are depicted in Table 1. We observe that all deep learning models (CNN, LSTM) clearly outperform shallow classifiers, which is explained by the greater representational and understanding power of the deep models in complex scenarios such as the discussed abnormal activity detection application. Moreover, the proposed approach based on optimized bidirectional LSTM attains higher performance rates compared to the other examined deep learning models, revealing the contribution of both the bidirectionality and the proposed framework for Bayesian optimization of the network parameters.

**Table 1.** Performance metrics on multimodal experiments

Method	Precision	Recall	Accuracy	F1 score
SVM-Linear	68.51%	61.71%	77.36%	64.93%
SVM-RBF	66.99%	60.06%	76.11%	63.34%
FNN1	69.95%	63.30%	78.52%	66.46%
FNN2	70.13%	63.50%	78.66%	66.65%
CNN	80.62%	76.09%	86.56%	78.29%
LSTM	81.14%	76.12%	87.11%	78.55%
Proposed Optimized Bidirectional LSTM	**90.01%**	**87.42%**	**88.70%**	**88.77%**

**Fig. 3.**
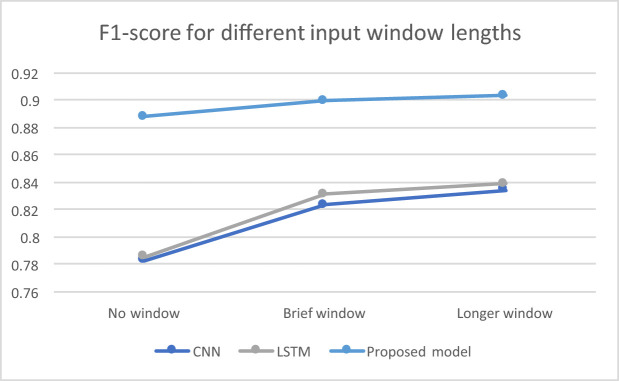
Attained F1-score for different input window lengths (number of frames) in the multimodal case for: (i) CNN, (ii) plain LSTM, and (iii) the proposed optimized bidirectional LSTM.

Finally, we have experimented with providing as input to the classifiers a “window” of past frames of different sizes, in other words feeding the model with “memory”. We have explored three cases for window length: no window, brief window (50 frames) and longer window (100 frames). The results for the multimodal case are depicted in Fig. 3. We can see that the presence of a time window in the input increases the performance in the examined cases of CNN, LSTM and the proposed optimized bidirectional LSTM, but the improvement ratio decreases as the window length increases. Furthermore, the improvement attained by the window is less significant in the proposed model compared to CNN and plain LSTM, where there is more room for improvement. In any case, though, the performance attained by the proposed model steadily outperforms the remaining examined approaches by a considerable difference.

## Conclusion

In this paper, we proposed a multimodal bidirectional Long Short-Term Memory neural network (LSTM) model for detection of abnormal activity in critical infrastructures. Three input modalities are considered: RGB, thermal and Channel State Information, the fusion of which is proved to provide significant added value in the unusual activity detection scenario. The multimodal input is fed into a bidirectional LSTM, which allows for an effective capturing of both forward and backward temporal dependencies. Moreover, a Bayesian optimization method is used to optimally select the parameters of the employed model. The presented methods have been experimentally evaluated with a real-world critical water infrastructure monitoring and protection dataset, and have been shown to achieve very promising detection rates.
